# Hypodermic Medication in Dental Practice

**Published:** 1870-07

**Authors:** W. F. Morrill


### HYPODERMIC MEDICATION IN DENTAL PRACTICE.

BY W. F. MORRILL.

Read at the meeting of the Indiana State Dental Association, June 28th, 1870.

In the practice of Dentistry a proper understanding of the healing art is not only indispensable but can scarcely be overestimated. A Dental practitioner who should fail to acquaint himself, in a tolerable degree, with this knowledge, would lay very sorry claims to professional rank, and in his practice would compliment his patients with skill very little beyond stupid quackery. Abundant sources of information, with respect to the science of materia medica, are at our hands, and if we neglect these opportunities for acquiring a fair share of proficiency in the action and use of medicines on the general economy, we shall be decidedly remiss, and very culpable indeed. It would seem that the constant embarrassment besetting the pathway of those engaged in practice, would be adequate provocation to insure a persistent and unremitting elevation to the science of materia medica.

In the study and cultivation of this specialty of our branch of medicine we find it most complex, because it investigates the laws of processes which are themselves departures from law. Ages of observation are before us; given to us by the greatest intellects, guided by a careful accuracy, and tested by the touchstone of experience. Brilliant theories have been furnished us; and what is of more significance to the welfare of the human race, splendid facts and results. Notwithstanding all this array of knowledge, coming down through the long corridors of centuries, we stand to-day merely at the threshold of the great domain of therapeutical science. Grand and beautiful as the achievements have been to the students of nature's divine mysteries, they have been able to discover some truths which marked the advancement of each succeeding age, and are permitted to see only the development of an insignificant portion of what is yet to be learned.

It is, then, the part of our duty, as it ought to be our pleasure, in the practice of our chosen specialty, that we keep pace with true progress in therapeutical knowledge, and each of us contribute a share towards its grand results.

The particular branch of therapeutics which I am invited to present to you at this meeting, is hypodermic medication. The introduction of this method is comparatively of recent date. M. Behier, a Frenchman, discovered, or originated it, and gave it popularity. Previous to his time, we had, and still have, numbers of methods of endermic medication—acupuncture, vesication, innoculation, are among the varieties employed—which attain similar results. Where diseases are of local nature, not very persistent, any of these methods possesses excellence and value. But the hypodermic method, on account of the ease and readiness with which it can be employed, renders it decidedly advantageous. The instruments used for this purpose consists of a small syringe, made of glass or rubber, holding about a drachm, graduated, or not, with a scale, and furnished with fine pointed, gold plated steel tubes of various sizes; which are thrust through the skin at an angle, while attached to the instrument. The medicines used are previously prepared in accurate solutions, and in administering them care should be observed while injecting the fluid into the tissue that the canula be withdrawn before the syringe is emptied of its entire contents. This precaution is deemed necessary on account of air, which might be forced into the circulation.

### ADVANTAGES.

The advantages of the introduction of medicines directly into the tissues of the system, instead of through the stomach, are chiefly the rapidity, intensity, and certainty of action which it secures. Moreover, the quantity of medicine employed is much reduced, more uniformity of action, and the time required to affect the system much less. The reasons for these advantages will be most apparent. The condition of the stomach from which absorption is dependent, may possess an irritability along the coatings, such as shall prevent the retaining of food or medicine. In the act of nausea and vomiting, it would be difficult in the extreme, where medicines have been introduced into the stomach and immediately rejected, to know whether any, all, or what quantity remains. Under such circumstances a repetition of doses, unless time be given, would be very hazardous. The quantity of food remaining in the stomach exercises a retarding influence and sometimes the gastric juices decompose the properties of medicine before absorption takes place.

It is also claimed by those who have much experience in hypodermic methods, that less nausea is likely to occur, less headache, less disturbance along the digestive apparatus; that it is more permanent, more prompt, uncomplicated, and decided; chiefly illustrated in the treatment of local disorders, especially in neuralgia of a particular nerve. It is an opinion generally accepted, that the administration of medicines into the stomach do not become anodynes where anodynes are intended for local pain, without affecting the whole economy. It is therefore recommended, when the object is to relieve local pain, the injection should be made near the seat of disease; due regard being had for the safety of the parts.

It is not my purpose to refer to or enter into any details with respect to the specific use and action of therapeutic agents, employed hypodermically, with which the medical publications can amply supply you; but rather to submit, briefly some suggestions pertinent to the introduction of this method in our own practice. There are some forms of disease which come to the notice of the dental practitioner that are proper for him to undertake the treatment of. Among these, seated within the maxillaries and contiguous parts we have dento-neuralgia, irritable pulps, pulpitis, pericementitis, and other disturbing pains, which are within the true province of the dental art to control. Dento-neuralgia, perhaps, presents the most difficult type, and baffles longest our best and well directed efforts before it will succumb. Among the narcotics chiefly used subcutaneously in proper doses, conjointly, and which produces unequivocal effect in the mastery of this disease are acetate of morphine and atropine. The more recent discovery of the hypnotic virtues of hydrate chloral, which are now being tried hypodermically, with much promise of success. Hydrate of chloral is represented, when admitted into the circulation, as becoming changed into chloroform, and producing very similar characteristic effects to that renowned narcotic. The blood is said to become, thereby, thinned, when chloral is used hypodermically, the temperature lowered, and a sleep of a protracted duration, lasting several hours.

If such are some of the patent virtues which hydrate of chloral possesses, it will certainly have decided advantage over the hitherto great sheet-anchor of materia medica, opium. And especially in inflammatory stages just begun, where the act of arrestation of blood corpuscles in the capillaries are impeded, this drug would experience a potent influence in promoting nutritive action by returning them to the circulation. This phenomena, called resolution, is certainly very desirable in diseased action, especially with threatening symptoms of alveolar abscess, unyielding to local treatment, if we can set bounds to it, neutralize the sthenic stage, or appease it, and control the formative action, we have accomplished one of the high missions of a skilled Dentist. Here doctors in medicine are not your equal. It is a pride and a triumph to be able to say to your patient, weary and worn with the agonies of pain: "Peace, be still." It lies not in the command, but in the obedience of your triumph. If you turn away the sufferers from your chairs unrelieved by your skill you incur not only their disapprobation, but a feeling of self-conscious guilt in professional incompetency, which is most grievous to be borne. The nature and variety of therapeutic agents are, at present, so well understood, and the appliances for their administration so ingenious and beautiful, that to become skilled in their use should be the noble ambition of every dental practitioner. But in administering medicinal substances directly into the circulating fluid, or blood, we should not lose sight of its important constituents. 'Let us see," says Professor Morton, most eloquently, "what this fluid is called upon to do, and so what sort of material it may require for its work. The blood is first to nourish and supply all parts of the complex fabric. It is to carry to the exhausted muscle material for its renewal; to it the bones, solid stone blocks, for building and repair; to it the throat, (that lute whose strings are always being worn out, and yet never out of tune) looks for its new cords; to it the eye looks for new lenses, and the ear for new fittings, for in those shell like chambers the nymph of harmony conceals herself. On its crimson tide float ever, whole navies of laden argosies, filled with most varied commodities. Food for all tissues, supplies for all organs, materials for all structures, form their various freight. Nor is this duty all. The same current must not only be the bearer of food and raiment to the inhabitants of its shores, but it must also be like the Ganges, their burial places. It must carry off the dead body of those cells which, having done their work, have by, and in that very act terminated their existence. It must be at once like the fertilizing Nile, strewing life along its shores; and like the river when stricken by the rod of Moses, a bloody river of death, hurry off the carcasses of its dead inhabitants to the sea."

